# Estimating DNA polymorphism from next generation sequencing data with high error rate by dual sequencing applications

**DOI:** 10.1186/1471-2164-14-535

**Published:** 2013-08-07

**Authors:** Ziwen He, Xinnian Li, Shaoping Ling, Yun-Xin Fu, Eric Hungate, Suhua Shi, Chung-I Wu

**Affiliations:** 1 State Key Laboratory of Biocontrol and Guangdong Key Laboratory of Plant Resources, Sun Yat-sen University, 135 Xingang West Road, Guangzhou 510275, China; 2 CAS Key Laboratory of Genome Sciences and Information, Beijing Institute of Genomics, Chinese Academy of Sciences, 1 Beichen West Road, Beijing 100101, China; 3 Human Genetics Center, University of Texas School of Public Health, 1200 Herman Presser Drive, Houston, TX 77030, USA; 4 Department of Ecology and Evolution, University of Chicago, 1101 East 57th Street, Chicago, IL 60637, USA

**Keywords:** Next generation sequencing, DNA polymorphism, Sequencing error, Pooled sample, Dual sequencing applications

## Abstract

**Background:**

As the error rate is high and the distribution of errors across sites is non-uniform in next generation sequencing (NGS) data, it has been a challenge to estimate DNA polymorphism (θ) accurately from NGS data.

**Results:**

By computer simulations, we compare the two methods of data acquisition - sequencing each diploid individual separately and sequencing the pooled sample. Under the current NGS error rate, sequencing each individual separately offers little advantage unless the coverage per individual is high (>20X). We hence propose a new method for estimating θ from pooled samples that have been subjected to two separate rounds of DNA sequencing. Since errors from the two sequencing applications are usually non-overlapping, it is possible to separate low frequency polymorphisms from sequencing errors. Simulation results show that the dual applications method is reliable even when the error rate is high and θ is low.

**Conclusions:**

In studies of natural populations where the sequencing coverage is usually modest (~2X per individual), the dual applications method on pooled samples should be a reasonable choice.

## Background

The next generation sequencing (NGS) technologies have dramatically increased the throughput. The new technologies, including those being developed currently, improve on many aspects of DNA sequencing but a higher accuracy than the traditional Sanger sequencing does not appear to be one of them. The nature of the technology would result in specific types of sequencing errors inherent in each process. In general, the new sequencing methods have an error rate between 0.1% and 1.0% [1]. Due to the non-random distribution of errors across sites where some sites can be 10 times more error prone than the average, single nucleotide polymorphism (SNP) calling can often be difficult [2-4].

In this study, we are concerned with estimating a fundamental parameter of natural populations, namely, Watterson's θ of DNA polymorphism [5]. Briefly, θ is the number of nucleotide differences between two sequences of the same locus, randomly chosen from the population. It is a good measure of genetic diversity and a basic parameter for doing population genetic analysis (e.g. tests of positive selection, [6-8]). As polymorphism in natural populations is dominated by low frequency variants [9], which are often indistinguishable from sequencing errors, using the new sequencing technologies to estimate polymorphism will remain a challenge in the near future. A number of methods have been proposed to separate errors from rare polymorphisms [10-14]. Among them, Nielsen *et al.*'s approach [14] is most direct by filtering out errors from the raw read data. However, since error signals may vary from operation to operation, its general applicability will need to be evaluated.

There are two ways to prepare samples for sequencing and polymorphism estimation. First, sequencing is done on individual samples, or at least on pooled samples with each sample individually barcoded [15]. We call this type of data “single-line data”. Second, DNA samples from multiple individuals are pooled in equal quantity for sequencing without individual identification [16]. It is referred to as “Pooled-line data”. We should note that sequencing each diploid sample individually is in fact a pooled-line approach as two haploid genomes are sequenced together. In order to call SNP accurately for both haploids, the diploid has to be sequenced to a sufficient depth (e.g. 20X) [3]. Since individual samples are generally not sequenced to such a depth (e.g. the 1000 Human Genome Project [17]), most methods cited above examine the aggregate properties of these individual sequences. In other words, although individuals may be sequenced separately, the data are pooled in the analysis. Hence, for many population genetic questions, little information would be lost by sequencing pooled samples and the efficiency would be greatly improved when the sample number is large. It would then be possible to sequence each pool with greater exactitude in order to filter out errors from the data.

We now propose a method which minimizes the confounding effects of sequencing errors by combining two different sequencing applications. Dual sequencing applications have previously been carried out on the Illumina GA and SOLiD platforms for the same samples [16,18,19]. It has been shown that the two technologies have nearly non-overlapping error distributions [4]. Dual platform is in fact a standard method as NGS sequencing, on whichever platform, needs to be backed up by another method, usually by Sanger sequencing or other genotyping tools [4,20,21]. Dual applications on two NGS platforms is simply a more systematic and large-scale method of error correction. Such dual applications can also be expected on newer and very different technologies such as HiSeq [22], Ion Protons [23], PacBio [24] and MspA nanopore [25]. When dual platform sequencing is not feasible, dual applications of the same platform on the same DNA sample, independently prepared for sequencing, may serve the same purpose. The correlation of error distribution between two applications on the same platform is slightly higher than those on different platforms but is often adequate for error corrections.

In this study, we first investigated a simple single-line method by extracting haploid information from individual diploids. We then propose dual sequencing applications to improve the pooled-line method for analyzing pooled samples of diploids.

## Results

### Single-line data

If the effort of data collection is not a limiting factor, the best method is to sequence each diploid individual to a sufficient depth such that true polymorphisms, with the variant frequency at 0.5, can be unambiguously separated from errors.

To ensure false positive error rate being less than 10%, it need more than 20X depth for most next generation sequencing platforms [3]. With a lower coverage, there would be many sites where the distinction between errors and polymorphisms is not possible. Therefore, when data are obtained with low coverage of diploid individuals (say, 2X), we suggest taking data from only one haploid genome per diploid individual. In this scheme, an average depth of 2X would ensure that 86% of individuals could be covered at each site, provided that the distribution of sequencing depth at each site follows a Poisson distribution, *p*(*depth* > 0) = 1-*e*^-2^. Since we are interested in comparing various methods of estimating genetic diversity, all of them are applied to data with an average depth of 2X per diploid individual.

#### Theory

Define θ as the nucleotide diversity per site. Let *S* denote the number of segregating sites and *l* denote the total number of sites. Watterson showed that

(1) $$E\left(S\right)=a_{n}\mathrm{\theta l},$$

where $a_{n}=\sum_{i=1}^{n-1}\frac{1}{i}$ and *n* is the sample size [26]. We assume *n* individuals with an average depth of 2X per individual. Hence, at site *j*, only *n*_*j*_ individuals would be sequenced (*n*_*j*_ ≤ *n*). Among these *n*_*j*_ individuals, we randomly select one read to represent a haploid genome of this individual. When this site is observed to be polymorphic among the *n*_*j*_ genomes, *S*_*j*_ = 1; otherwise, *S*_*j*_ = 0. In the absence of sequencing error, the estimate of θ is

(2) $$\hat{\theta }=\frac{1}{l}\sum_{j=1}^{l}\frac{S_{j}}{a_{n_{j}}},$$

where $a_{n_{j}}=\sum_{i=1}^{n_{j}-1}\frac{1}{i}$.

Because some variants observed among the *n*_*j*_ individuals would be sequencing errors, we need to consider a more reliable portion of the frequency spectrum in the estimation of θ. Given the current sequencing error rate [1], sequencing errors would usually appear as singletons (number of variant, *b*, being 1 or *n*_*j*_-1) or doubletons (*b* = 2,*n*_*j*_-2). Ewens showed that $\frac{1}{b}/\sum_{i=1}^{n-1}\frac{1}{i}$ is the probability that a mutant is represented *b* times in *n* samples and the estimate of θ should be

(3) $$\hat{\theta }=\frac{1}{l}\sum_{j=1}^{l}\frac{S_{j}}{{a^{\prime }}_{n_{j}}},$$

where ${a^{\prime }}_{n_{j}}=\sum_{i=1+z}^{n_{j}-1-z}\frac{1}{i}$[9]. In this formula, *z* = 1 when singletons are removed and *z* = 2 when both singletons and doubletons are removed.

#### Simulations

We simulated a 100 kb region of different θ and sequencing error rate. The results are presented below the heading of “Single-line” in Table 1. *S*_>0_ denotes that all segregating sites detected by reads are counted. *S*_>1_ represents all segregating sites excluding singletons, while *S*_>2_ excluding both singletons and doubletons.

**Table 1:** Estimating θ with constant sequencing error rate

**Error rate**	**Sites used**	**θ = 0.1 / kb**			**θ = 1 / kb**		
		**Single line**	**Pooled-lines**		**Single line**	**Pooled-lines**	
			**single platform**	**dual applications**		**single platform**	**dual applications**
	S_>0_	0.099 (0.007)	0.100 (0.003)	0.100 (0.007)	0.999 (0.023)	1.000 (0.009)	0.999 (0.022)
0	S_>1_	0.100 (0.012)	0.100 (0.005)	0.100 (0.010)	0.999 (0.041)	1.000 (0.016)	1.000 (0.034)
	S_>2_	0.099 (0.016)	0.100 (0.007)	0.100 (0.013)	0.998 (0.050)	1.000 (0.023)	1.000 (0.041)
	S_>0_	5.992 (0.131)	22.054 (0.212)	0.323 (0.026)	6.884 (0.131)	22.872 (0.225)	1.226 (0.033)
0.001	S_>1_	0.129 (0.017)	0.507 (0.032)	0.100 (0.011)	1.032 (0.040)	1.409 (0.035)	1.003 (0.034)
	S_>2_	0.101 (0.017)	0.105 (0.008)	0.100 (0.013)	0.999 (0.052)	1.009 (0.023)	1.002 (0.042)
	S_>0_	28.389 (0.271)	90.901 (0.357)	5.269 (0.116)	29.184 (0.275)	91.485 (0.358)	6.165 (0.118)
0.005	S_>1_	0.810 (0.054)	9.295 (0.149)	0.112 (0.012)	1.716 (0.064)	10.167 (0.152)	1.024 (0.035)
	S_>2_	0.112 (0.017)	0.662 (0.039)	0.100 (0.014)	1.016 (0.053)	1.575 (0.046)	1.010 (0.042)
	S_>0_	53.883 (0.351)	146.007 (0.357)	18.820 (0.215)	54.615 (0.331)	146.329 (1.111)	19.684 (0.218)
0.01	S_>1_	2.861 (0.105)	31.994 (0.258)	0.257 (0.025)	3.777 (0.107)	32.823 (0.821)	1.179 (0.040)
	S_>2_	0.180 (0.027)	4.061 (0.108)	0.102 (0.013)	1.091 (0.057)	4.993 (0.327)	1.017 (0.041)

The average depth is 2X per individual in single line method, 2X per haploid genome in single platform method and 1X per haploid genome in each application in dual applications method. The means (and the standard deviations) of θ are estimated from 1000 replicates. Error rate is per site.

When the error rate is set to 0, the estimates of θ using *S*_>0_ are very close to the true values. When the error rate is 0.001 to 0.01, the estimates of θ using *S*_>0_ become extremely unreliable, as expected, and the removal of singletons and doubletons becomes necessary. With an error rate of 0.001, the estimate of θ using *S*_>2_ is 0.101, very close to the true value of 0.1. If the error rate is as high as 0.01, estimation by the single line method becomes unreliable even the singletons and doubletons are removed.

A serious problem in SNP calling is the non-random distribution of errors across sites [2,3]. In reality, some sites can be 10 times more error prone than the rest [4]. We hence conducted simulations with the assumption that the error rate is Beta distributed (*ϵ* ~ *Beta*(*a*,*β*)). We use different shape parameters (*a* = 0.1,0.2,0.4 *and* 0.8). It is clear from Table 2 that, when the error rate is non-constant, the single line method is not accurate for estimating θ even with the removal of singletons and doubletons.

**Table 2:** Estimating θ with Beta distributed sequencing error rate

**Parameter α of Beta distribution**	**Sites used**	**θ = 0.1 / kb**			**θ = 1 / kb**		
		**Single-line**	**Pooled-lines**		**Single-line**	**Pooled-lines**	
			**single platform**	**dual applications**		**single platform**	**dual applications**
	S_>0_	19.917 (0.237)	38.335 (0.274)	2.534 (0.083)	20.759 (0.227)	39.095 (0.267)	3.444 (0.085)
0.1	S_>1_	4.709 (0.139)	17.500 (0.197)	0.336 (0.032)	5.605 (0.133)	18.347 (0.197))	1.248 (0.043)
	S_>2_	1.172 (0.073)	9.541 (0.159)	0.130 (0.017)	2.073 (0.081)	10.425 (0.156)	1.041 (0.042)
	S_>0_	23.170 (0.255)	51.539 (0.290)	3.499 (0.098)	23.964 (0.230)	52.237 (0.306)	4.393 (0.097)
0.2	S_>1_	3.343 (0.112)	18.415 (0.203)	0.250 (0.026)	4.243 (0.120)	19.249 (0.208)	1.160 (0.040)
	S_>2_	0.534 (0.049)	7.555 (0.143)	0.109 (0.014)	1.443 (0.071)	8.444 (0.145)	1.015 (0.041)
	S_>0_	25.398 (0.240)	64.217 (0.333)	4.243 (0.105)	26.210 (0.239)	64.855 (0.335)	5.134 (0.108)
0.4	S_>1_	2.259 (0.095)	17.193 (0.201)	0.181 (0.019)	3.164 (0.097)	18.017 (0.201)	1.090 (0.038)
	S_>2_	0.267 (0.034)	4.916 (0.114)	0.103 (0.013)	1.175 (0.059)	5.808 (0.114)	1.010 (0.042)
	S_>0_	26.772 (0.270)	74.504 (0.355)	4.717 (0.112)	27.578 (0.262)	75.097 (0.340)	5.609 (0.111)
0.8	S_>1_	1.591 (0.073)	14.860 (0.196)	0.143 (0.015)	2.492 (0.087)	15.706 (0.181)	1.054 (0.035)
	S_>2_	0.171 (0.024)	2.870 (0.084)	0.101 (0.013)	1.076 (0.057)	3.773 (0.088)	1.010 (0.041)

The average error rate is 0.005 per site. The average depth is 2X per individual in single line method, 2X per haploid genome in single platform method and 1X per haploid genome in each application in dual applications method. The means (and the standard deviations) of θ are estimated from 1000 replicates.

### Pooled-lines data from single platform

From the section above, it appears that the most efficient strategy for accurately estimating genetic diversity would not be single-line sequencing. Given the low coverage for each individual, variant frequencies, rather than the genotypes of individuals, are the quantities of interest. Pooling samples for bulk sequencing may be equally informative but at a lower cost and effort [16]. When pooled samples are sequenced, each haploid genome would not present equally in the final data and the coverage would vary from site to site. The statistics to correct for these fluctuations are given below. In this and the next sections, the pooled samples are sequenced by one single application or by dual applications. The ability to separate errors from true polymorphisms differs greatly between the two approaches.

#### Theory

Equal amount of DNAs from each individual are pooled and the pooled samples are sequenced on one sequencing platform. Assuming a segregating site with *b* mutants in a sample of size *n* is covered by *r* reads in an Illumina GA or SOLiD dataset, Jiang *et al.*[10] showed that the probability *q*_1_(*b*) that this segregating site is detected by reads is

(4) $$q_{1}\left(b,r\right)=1-{\left(1-b/n\right)}^{r}-{\left(b/n\right)}^{r},$$

for 0 < *b* < *n*, and the probability *q*_2_ that a segregating site with an arbitrary *b* value is detected by reads is

(5) $$q_{2}\left(r\right)=\sum_{b=1}^{n-1}q_{\mathrm{nb}}q_{1}\left(b,r\right).$$

Ewens showed that $q_{\mathrm{nb}}=\left(1/b\right)/a_{n}=\frac{1}{b}/\sum_{i=1}^{n-1}\frac{1}{i}$ is the probability that a mutant presents *b* times in *n* samples [9].

Let *S*_*T*_ denote the number of segregating sites detected by reads, and we can obtain

(6) $$E\left(S_{T}\right)=\frac{S}{l}\sum_{j=1}^{l}q_{2}\left(r_{j}\right),$$

where *r*_*j*_ is the number of reads covering the site *j*. Hence the estimate of θ is

(7) $$\begin{array}{l} \hat{\theta }=S/a_{n}l\\ \;=\frac{E\left(S_{T}\right)}{a_{n}\sum_{j=1}^{l}q_{2}\left(r_{j}\right)}. \end{array}$$

Replacing *q*_2_ with equation (5) yields

(8) $$\hat{\theta }=\frac{E\left(S_{T}\right)}{\sum_{j=1}^{l}\sum_{b=1}^{n-1}\frac{q_{1}\left(b,r_{j}\right)}{b}}.$$

Now we shall consider a more realistic case with sequencing errors in the data. Let’s assume a case in which a site is covered by *r* reads in a single platform and has mismatches in *x* read(s) caused by sequencing error. The probability *P*_*ϵ*_(*r,x*) of its occurrence at a non-segregating site is

(9) $$p_{\epsilon }\left(r,x\right)=C_{r}^{x}\epsilon^{x}{\left(1-\epsilon \right)}^{r-x},$$

where *ϵ* denotes the sequencing error at this site. Since the average raw error rate ranges from 0.1% to 1.0% [1], the sequencing error can cause severe problems when estimating polymorphism.

However, if using an observed segregating site only when the minor allele has more than *z* reads, we may obtain more accurate estimates. Instead of equation (4), the probability that a site with *b* mutants in a sample of size *n* is detected by *r* reads as a segregating site with more than *z* reads of each allele is

(10) $$\begin{array}{ll} \;q_{1}\left(z,b,r\right)= & 1-\sum_{x=0}^{z}C_{r}^{x}{\left(1-b/n\right)}^{r-x}{\left(b/n\right)}^{x}\\ & -\sum_{x=r-z}^{r}C_{r}^{x}{\left(1-b/n\right)}^{r-x}{\left(b/n\right)}^{x}. \end{array}$$

The estimate of θ is now

(11) $$\hat{\theta }=\frac{E\left(S_{>z}\right)}{\sum_{j=1}^{l}\sum_{b=1}^{n-1}\frac{q_{1}\left(z,b,r_{j}\right)}{b}}.$$

*S*_>*z*_ denotes the number of segregating sites at which two different alleles are both detected by more than *z* reads. All segregating sites detected by reads are counted when *z* = 0. Hence, *S*_>0_ is equal to *S*_*T*_.

The procedure to estimate θ using pooled-lines data from single platform is as follows. For each site, we (1) treat the data as missing if the number of reads is less than *r*_min_ in this platform; (2) retain alleles having more than *z* reads. If there is only one allele in this platform, we treat this site as a nonsegregating site; if two, as a segregating site; if more than two, we treat the data as missing; (3) use equation (11) to calculate θ for the single platform. *r*_min_ should not be no lower than (2*z* + 2). In the following simulations, we set *r*_min_ = 6.

#### Simulations

We used the simulated data to test this method. The results are referred to as “single platform” in Table 1. When singletons (the minor depth allele is covered by only one read, *z* = 1) or both singletons and doubletons (the minor depth allele is covered by two reads, *z* = 2) are discarded (the row “*S*_>1_” and “*S*_>2_”), the standard deviation becomes larger if there is no sequencing error. In reality, sequencing error cannot be ignored.

We assume that the error rate is constant across sites. Different error rates (0.001, 0.005 and 0.01) are used in the estimation. The simulation results are displayed in Table 1. For example, when *S*_>0_ is used with an error rate of 0.001, sequencing errors lead to very poor estimates of θ. The mean estimate of θ is 22.054 per kb if all segregating sites are used, which is many times higher than the true value of 0.1 per kb. The estimation becomes more accurate when *S*_>1_ or *S*_>2_ is used. Thus, when the error rate is low (e.g. 0.001), this method can be used to estimate θ with both singletons and doubletons discarded. However, when the error rate is high (e.g. 0.005 or 0.01), even excluding singletons and doubletons (the row “*S*_>2_” in Table 1) does not lead to acceptable estimates. For simulations with the assumption that the error rate is Beta distributed and its mean is 0.005, the estimates are also unacceptable (shown in Table 2).

### Pooled-lines data from dual sequencing applications

It is customary to validate calls of variants by another method. For example, variant calls on the Illumina platform are often validated by Sanger sequencing or by fast SNP genotyping methods, e.g. Sequenom genotyping [4,20]. Because validation is often laborious and incomplete, it may be more efficient and informative to deploy two sequencing methods fully and independently. If the two applications have distinctive error-distribution patterns, the errors could be identified and excluded by reciprocally correcting each other’s errors. Indeed, several widely used sequencing methods (as well as the latest methods that are in development) are based on very different chemistry and protocols. As shown below, we analyzed the sequencing results obtained by Illumina based data and SOLiD, and as expected, we observed the two datasets showed non-overlapping errors.

#### Data on error correlation between sequencing applications

**Figure 1:**
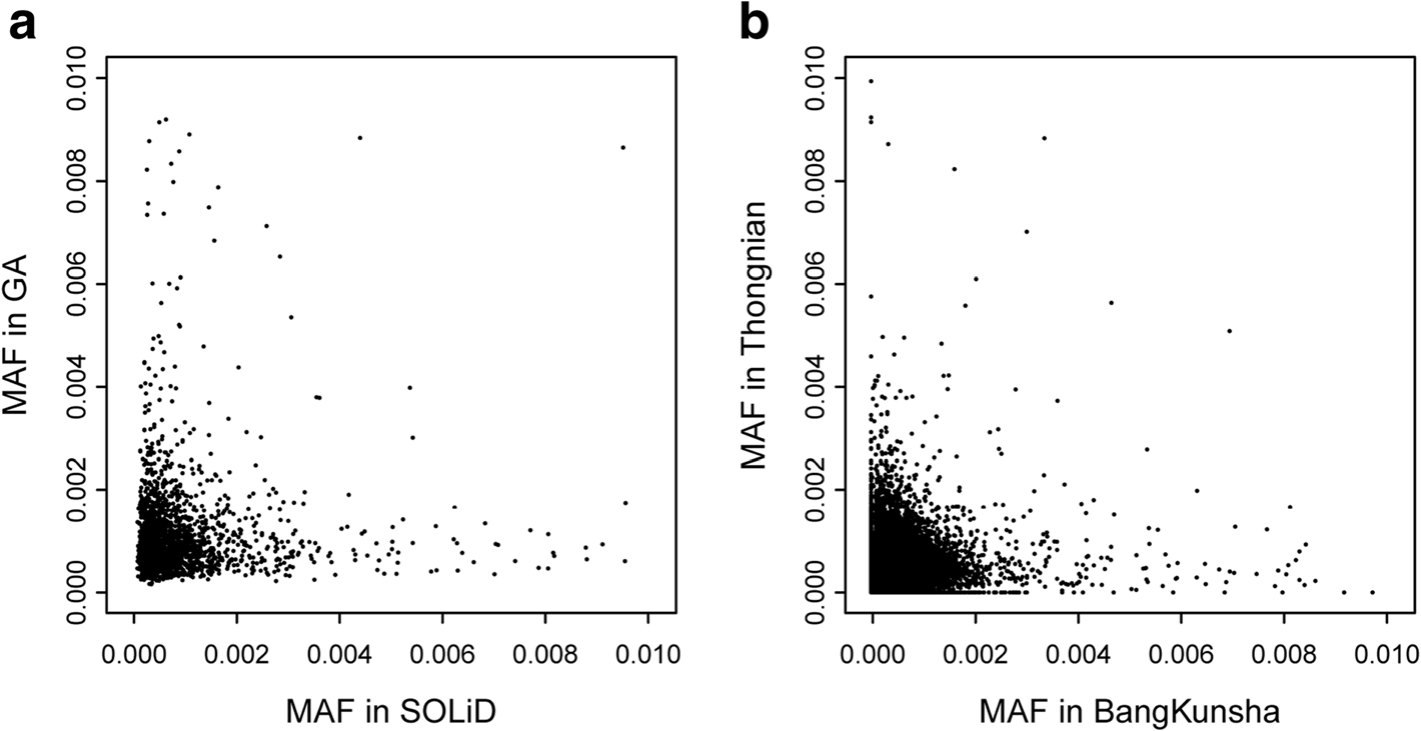
**Error rate correlation patterns. a)** MAF (minor allele frequency) of putative SNPs called by either SOLiD or Illumina GA. **b)** MAF in two samples (Bangkunsha and Thongnian) sequenced by Illumina HiSeq.

*Dual platforms* - We re-analyzed sequencing data from a species of mangrove trees, *Sonneratia alba*, known to be completely monomorphic within some populations [4]. DNA sequences for 71 genes from one such population were generated using the Illumina GA and SOLiD platforms at a depth of ~2500X and ~5400X, respectively. For sites with more than 2000X depth in both platforms, we called variants using a set of criteria more stringent than the previous study. As shown in Figure 1a, Illumina GA and SOLiD systems both call many false SNPs, few of which are called by both. Because the sample is known to be monomorphic by Sanger sequencing [4], the detected variants are all false SNPs, which fortunately do not show overlap between platforms. Pearson's correlation coefficient of the error rate distributions between the two platforms is only 0.054.

*Single platform* - For analyzing the correlation of two samples sequenced on the same platform, we use our own unpublished data from the Illumina HiSeq platform. A sample of 35 individuals from a mangrove species, *Avicennia marina,* was taken from each of two nearby populations in Thailand. Equal amount of DNAs from 35 individuals (or 70 haploid genomes) were pooled. 93 genes were amplified for both of the two pooled samples and sequenced on an Illumina HiSeq platform. For sites with more than 2000X depth in both samples, we called SNPs at the sites whose minor allele frequency (MAF) is lower than 0.01 in both samples. In total, 55,602 sites were retained and were plotted in Figure 1b. Almost all of these variants are sequencing errors as explained in Methods. Figure 1b shows the observed error rates on these sites. Pearson's correlation coefficient of the error rate distributions between these two samples is only 0.142, a little higher than that between platforms of Figure 1a. Therefore, for samples prepared and sequenced twice on one platform, sequencing errors also overlap only rarely.

#### Theory

If sequencing errors from two applications do not overlap, segregating sites detected by both should be true variants. The probability *q*_1_(*b*) that a segregating site with *b* mutants in a sample of size *n* is detected in both applications is

(12) $$q_{1}\left(b,r_{1},r_{2}\right)=\prod_{k=1,2}\left(1-{\left(1-b/n\right)}^{r_{k}}-{\left(b/n\right)}^{r_{k}}\right).$$

The overall estimate of θ by the combined data is

(13) $$\hat{\theta }=\frac{E\left(S_{T}\right)}{\sum_{j=1}^{l}\sum_{b=1}^{n-1}\frac{q_{1}\left(b,r_{1}{}_{j},r_{2}{}_{j}\right)}{b}},$$

where *r*_1*j*_ is the number of reads covering site *j* in the first dataset, while *r*_2*j*_ is the number of reads covering site *j* in the second dataset.

For non-overlapping errors, a site with *b* mutants in a sample of size *n* that is detected as a segregating site with more than *z* reads of each allele in both applications is associated with the probability

(14) $$\begin{array}{ll} \;q_{1}\left(z,b,r_{1},r_{2}\right)=\prod_{k=1,2} & (1-\sum_{x=0}^{z}C_{r_{k}}^{x}{\left(1-b/n\right)}^{r_{k}-x}{\left(b/n\right)}^{x}\\ & -\sum_{x=r_{k}-z}^{r_{k}}C_{r_{k}}^{x}{\left(1-b/n\right)}^{r_{k}-x}{\left(b/n\right)}^{x}). \end{array}$$

The θ estimated by the dual applications method is

(15) $$\hat{\theta }=\frac{E\left(S_{>z}\right)}{\sum_{j=1}^{l}\sum_{b=1}^{n-1}\frac{q_{1}\left(z,b,r_{1}{}_{j},r_{2}{}_{j}\right)}{b}}.$$

Here *S*_>*z*_ denotes the number of segregating sites in which two different alleles are both detected by more than *z* reads on both applications.

The procedure to estimate θ using data from dual sequencing applications is as follows. For each site, we (1) treat the data as missing if the number of reads is less than *r*_min_ on either applications; (2) retain alleles having more than *z* reads on both applications. If there is only one allele on either application, we treat this site as a non-segregating site. A site is considered segregating only when reads from both applications report segregation; (3) use equation (15) to calculate θ for the combined dataset. We set *r*_min_ = 6 in the following simulations.

#### Simulations

**Figure 2:**
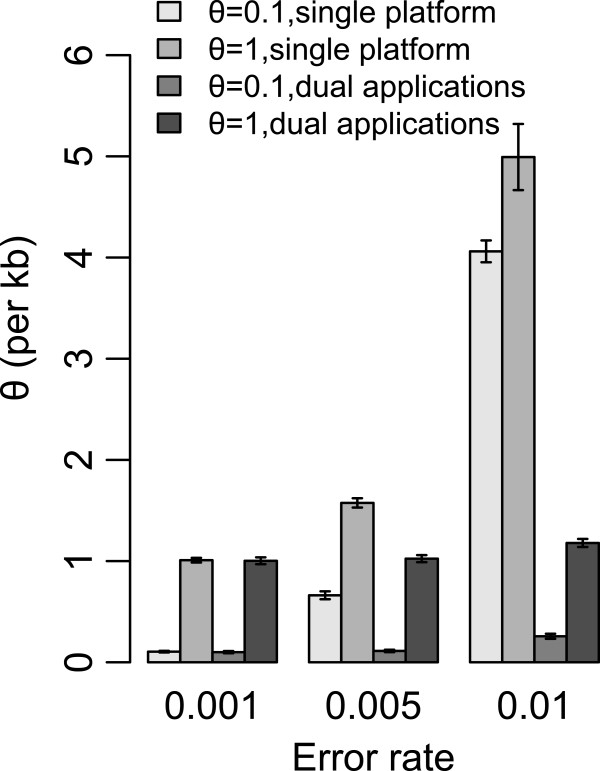
**θ estimation of simulation data of pooled-lines sample with 3 different sequencing errors.** The θ value of simulation data is set to 0.1 / 1 per kb. Singletons are discarded in dual applications method (S_>1_). Singletons and doubletons are discarded in single platform method (S_>2_). The length of each error bar is 2 times the standard deviation. The means (and the standard deviations) of θ are estimated from 1000 replicates.

The simulation procedure is almost the same as that for the single platform, but with data from an additional sequencing application. The means and the standard deviations of θ estimates using different parameters are reported in Table 1. For sequencing data without errors, the dual platform method can accurately estimates θ, although the standard deviation values are slightly larger than those obtained by the single platform method. However, with the increase of the error rate, the advantage of the dual platform method compared with other methods becomes obvious (Figure 2). With an error rate of 0.01, the mean estimate of θ is 0.102 per kb when using *S*_>2_, which is only 2% higher than the real value (0.1 per kb). This estimate is dramatically better than the corresponding single platform estimate (4.061) or the single line estimate (0.180). This method is also better than the others when the error rate is Beta distributed as shown in Table 2.

**Figure 3:**
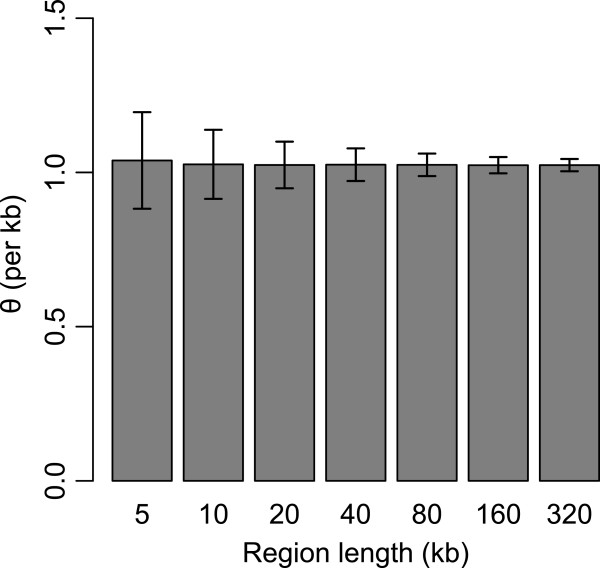
**θ estimation of dual applications for different region length.** The θ value of simulation data is set to 1 per kb. The sequencing error rate is set to 0.005. Singletons are discarded in the estimation (S_>1_). The length of each error bar is 2 times the standard deviation. The means (and the standard deviations) of θ are estimated from 1000 replicates.

In Figure 3, we used different region lengths to test the dual applications method. The estimations of θ is acceptable even when the region is small (e.g. 10 kb). For a 40 kb region (the real number of S is about 180), the standard deviation of θ estimates is account for only 5% of the real θ.

## Discussion

While NGS has increased the power of DNA sequencing by orders of magnitude in the recent years, its accuracy per read is the one aspect that has not been improved. For example, 454 Pyrosequencing is susceptible to homopolymer indels [1]. The Illumina GA and SOLiD platforms are both PCR based systems and are prone to base substitution errors. The first glimpses of newer technologies do not offer promises for improving per read accuracy either. Nevertheless, the nature of the substitution errors may differ among platforms since major sources of errors, from library construction to base-pair determination, depend on different physical and chemical principles among these technologies. The method described herein takes advantage of the non-overlapping distributions to minimize error rates.

The error rate across all sites is platform-dependent and not constant (e.g. Beta distribution) [4]. When doing the simulation, we assume that a nucleotide has an equal probability of being read incorrectly as one of the three other nucleotides. However, the patterns of error rates for the real data are much more complex. The frequencies of base substitution error could vary by 10 to 11 fold, with A to C transversions being among the most frequent substitution errors and C to G transversions among the least frequent ones [27]. Therefore, if a non-segregating site (e.g. A) has two reads with sequencing errors, a doubleton error is more likely (e.g. two A to C errors) rather than two singleton errors (e.g. one from A to C and another from A to T). In other words, the unevenly distributed errors can cause severe problems in estimating polymorphism. In this situation, we strongly suggest using dual sequencing applications to avoid this kind of errors.

## Conclusions

Our model can estimate θ accurately by combining data from two different sequencing applications. The method is robust even when the error rate is extremely high and variable across sites. We also evaluated the relative merits of pooled-lines versus single-line data. If the coverage per line is low, dual sequencing application on pooled lines yields the best results. However, the inherent high error rates in the NGS technologies impose constraints on the estimation of polymorphisms. Even under the best of conditions with sequencing done on two platforms, singletons and doubletons still have to be removed. If the estimation requires accuracy in the low frequency portion of the variant spectrum, it will be necessary to carry out sequencing on each line individually with a high coverage of >20X. For many scientific questions, our strategy of dual sequencing applications on pooled samples with modest coverage can yield the most information for the same level of effort.

## Methods

### Sample preparing and sequencing

We sampled two *Avicennia marina* populations (Bangkunsha and Thongnian) in Thailand. Equal amount of DNAs from 35 individuals (diploids) in each population were pooled, respectively. 93 genes were respectively amplified for both of the two pooled samples and then sequenced on an Illumina HiSeq platform.

### Reads alignment and SNPs calling

We use *MAQ*[28] to align reads to the known references. Nucleotides with base quality low than 20 are discussed. For sites with more than 2000X depth in both samples, we called candidate polymorphic sites whose minor allele frequency is lower than 0.01 in both samples. In total, 55,602 sites were retained and were plotted in Figure 1b.

In re-analyzing sequencing data of *Sonneratia alba,* Singletons are discarded and only the mutant alleles with at least one read aligned in forward strands and one read aligned in backward strands are retained for the following analyses. 2382 candidate sites were plotted in Figure 1a.

### Searching sites with errors

Consider a singleton site with MAF being 1/70, if the sequencing depth of this site is 2000X, we can infer the probability of observing its MAF < 0.01 to be 0.0375, using the distribution function of a Binomial distribution. For a singleton with the same MAF being observed in both samples, the probability is 0.0014 (the square of 0.0375). If the site has more than two mutant alleles or the depth is more than 2000X, the probability will decrease. The total number of SNPs of these two populations is estimated no more than 500 for these 93 genes. Therefore, there should be no more than 0.7 (0.0014*500) true polymorphic sites in Figure 1b. Near all candidate polymorphic sites in Figure 1b are introduced by errors.

### Simulation progress

We simulated sequencing progress with a Poisson distributed depth. Errors were added randomly for each site with the given error rate. We wrote Perl scripts to evaluate θ for single/dual applications method described in the main text. The means and the standard deviations of θ for each combination of parameters in Table 1 and Table 2 are estimated from 1000 replicates.

For single-line data, we simulated a 100 kb region for 25 diploid individuals with an average depth of 2X; hence, *Max*(*n*_*j*_) = 25 as only one read is used per individual. We set θ to be 0.1 or 1 per kb and error rate to be 0, 0.001, 0.005 or 0.01 per site.

For pooled-lines data, a 100 kb region is simulated for 25 diploids (50 haploid genomes) using a single platform or dual platforms. We set different θ values (0.1 / 1 per kb) and used *S*_>0_, *S*_>1_ and *S*_>2_ in the estimate. The average depth is 2X per haploid genome in single platform method and 1X per haploid genome in each application in dual applications method.

## Abbreviations

NGS: Next generation sequencing; SNP: Single nucleotide polymorphism; MAF: Minor allele frequency.

## Competing interests

The authors declare that they have no competing interests.

## Authors’ contributions

ZH carried out the theoretical derivation, simulation and drafted the manuscript. XL and SL carried out the sequence alignment and the analysis of the correlation of sequencing errors between two platforms/samples. YXF participated in the theoretical derivation. EH participated in the simulation and helped to draft the manuscript. SS and CIW conceived using dual sequencing applications to correct sequencing errors and helped to draft the manuscript. All authors read and approved the final manuscript.
